# Drainage of a Tense Propofol Extravasation Through a Pre-existing Intravenous Tract in an Adolescent: A Case Report

**DOI:** 10.7759/cureus.115949

**Published:** 2026-09-08

**Authors:** Paolo Taffache

**Affiliations:** 1 General and Pediatric Anesthesia and Intensive Care Unit, Istituto di Ricovero e Cura a Carattere Scientifico (IRCCS) Azienda Ospedaliero Universitaria (AOU) Bologna-Policlinico di Sant'Orsola, Bologna, ITA

**Keywords:** compartment syndrome, decompression, pediatric anesthesia, propofol extravasation, tissue ischemia

## Abstract

Propofol extravasation is often self-limited but can cause tissue necrosis and, rarely, compartment syndrome. A healthy 13-year-old girl (35 kg) undergoing hysteroscopy developed extravasation at the antecubital fossa during the manual administration of 10 mL of propofol 1% through a previously placed 24-gauge peripheral intravenous catheter in the right cephalic vein. A 5-mL saline flush immediately beforehand caused neither pain nor visible swelling. Near the completion of the propofol administration, pain developed with marked swelling, pallor, and capillary refill of approximately four seconds; the radial pulse and hand motor and sensory function remained intact. The patient remained fully conscious and responsive without evident systemic sedative effects. Propofol administration was stopped, another intravenous line was established, and general anesthesia was induced through the new access. The actual amount of the initial 100-mg propofol dose that extravasated into the tissues could not be determined. Aspiration through the original catheter yielded no return. Five 22-gauge passive drainage punctures and subsequent aspiration and enlargement of the catheter tract with a 16-gauge needle also produced no drainage.

A pediatric surgeon who was present suggested limited blunt opening of the pre-existing catheter tract. A small Klemmer clamp was introduced approximately 5 mm into the tract and gently opened in multiple radial directions, allowing the immediate drainage of a clinically estimated 8-10 mL of whitish lipid-containing fluid mixed with blood, followed by rapid tissue softening and improvement in local perfusion. Formal ultrasonography at approximately 24 hours showed soft-tissue edema without a significant fluid collection, and clinical recovery was complete at seven days. This case describes drainage through a pre-existing intravenous tract after failed catheter aspiration and needle drainage. The maneuver was nonstandard, cannot be considered an alternative to guideline-directed surgical assessment or washout, and should not delay urgent surgical evaluation when compartment syndrome or ongoing neurovascular compromise is suspected. This single-case observation is hypothesis-generating and does not support the routine adoption of the maneuver.

## Introduction

Propofol extravasation has traditionally been considered less locally injurious than the extravasation of classic vesicants, in part because standard lipid-emulsion formulations are approximately isotonic and near neutral in pH. Nevertheless, clinically important injury has been reported, including skin and soft-tissue necrosis in adults and children and rare acute compartment syndrome requiring fasciotomy [1-7]. A 2023 review proposed an “absorption-refractory” mechanism for propofol and lipids, in which retained extravasation may persist in tissue, while pediatric guidance notes that extravasated volume can contribute to compressive injury and compartment syndrome [8,9].

The standard early management of extravasation includes the immediate cessation of administration, leaving the intravenous catheter in place initially for attempted aspiration, avoiding further flushing, elevation when appropriate, and serial neurovascular assessment [9]. Pediatric guidance identifies blanching and prolonged capillary refill as signs of impending tissue ischemia and considers neurovascular compromise or suspected compartment syndrome a surgical emergency [9].

This report describes an adolescent with a tense antecubital propofol extravasation and early perfusion compromise in whom catheter aspiration and percutaneous drainage failed, followed by rapid drainage after limited blunt opening of the pre-existing intravenous tract. This single-case observation is intended to be hypothesis-generating and should not be interpreted as advocating this maneuver as a standard or validated treatment.

## Case presentation

A healthy 13-year-old girl (150 cm; 35 kg; American Society of Anesthesiologists physical status I) was scheduled for hysteroscopy under general anesthesia. On arrival in the pediatric operating room, she had a 24-gauge peripheral intravenous catheter that had previously been placed on the ward in the right cephalic vein at the antecubital fossa. The line had been clamped during transfer. Immediately before induction, 5 mL of 0.9% sodium chloride was injected manually through the catheter. The patient reported no pain, and no visible swelling occurred. The flush therefore appeared clinically normal, although secure intravascular position of the catheter tip could not be independently confirmed.

Propofol 1% was then administered manually from a 20-mL syringe. A total of 10 mL (100 mg; 2.86 mg/kg) was administered through the catheter. The actual amount that extravasated into the tissues could not subsequently be determined. No distinct increase in injection resistance was appreciated through the 24-gauge catheter. Near the completion of the administration, the patient reported pain at the injection site, coinciding with the rapid development of marked swelling and tension around the antecubital fossa. The overlying skin became clearly pale compared with the surrounding tissue, and capillary refill in the affected limb was approximately four seconds. The radial pulse remained palpable, and hand motor function and sensation were preserved. Propofol administration was immediately stopped.

At the time the extravasation was recognized, the patient remained fully conscious and responsive, with no evident somnolence, loss of consciousness, or other clinically apparent systemic sedative effect from the initial propofol administration. She was agitated and in pain. Before induction through a newly established intravenous access, her heart rate was approximately 120 beats/minute, blood pressure was 130/80 mmHg, and oxygen saturation (SpO₂) was 100%.

A new peripheral intravenous catheter was promptly placed at another site. A further 100 mg of propofol was administered intravenously through the new access to induce general anesthesia. No clinically relevant hemodynamic or respiratory complications occurred during or immediately after the repeat induction. Further local interventions were performed while the patient was anesthetized.

Aspiration through the original 24-gauge catheter with an empty syringe yielded no blood, fluid, or visible propofol. Five 22-gauge needles were then inserted percutaneously into the areas of greatest tissue tension to an approximate depth of 1 cm. These needles were used solely to provide passive drainage pathways and were not connected to syringes; no negative-pressure aspiration was applied. No fluid was obtained, and the degree of swelling and tissue tension remained unchanged.

The 24-gauge catheter was then removed. A 16-gauge needle was introduced through the pre-existing puncture tract to an approximate depth of 3 cm. Negative-pressure aspiration through the 16-gauge needle yielded no fluid. After the removal of the needle, the enlarged tract was observed for passive drainage without the application of external pressure, but no fluid emerged, and the swelling and tissue tension remained unchanged.

A pediatric surgeon was present during the event and, with marked tissue tension, pallor, and delayed capillary refill persisting despite failed aspiration and needle drainage, suggested limited blunt opening of the pre-existing catheter tract. Following this recommendation, a small Klemmer clamp was introduced through the same tract to an approximate depth of 5 mm. The jaws were gently opened in multiple radial directions to produce limited blunt spreading. Whitish, oily-appearing fluid mixed with blood, visually consistent with extravasated propofol emulsion, immediately began to drain. A gentle external expression of the swollen tissues facilitated evacuation. The recovered volume was not formally measured but was clinically estimated at approximately 8-10 mL; this estimate included blood and tissue fluid and therefore does not represent a measured volume of extravasated propofol.

The response was immediate: The previously tense tissues became soft, pallor resolved, and distal perfusion improved. The interval from the recognition of the extravasation to the successful drainage maneuver was less than 10 minutes. Capillary refill subsequently remained approximately two seconds, and no recurrent tissue tension developed. No significant bleeding or immediately apparent neurovascular complication occurred following the maneuver.

At approximately 24 hours, a formal ultrasonographic examination was performed using a high-frequency linear transducer. The examination demonstrated soft-tissue edema without a significant residual fluid collection. Neither color nor power Doppler interrogation was performed. Patency or thrombosis of the cephalic and basilic veins was not specifically documented in the report, and a dedicated assessment of the deeper fascial compartments was not documented. The ultrasound examination should therefore be interpreted primarily as a follow-up assessment of soft-tissue edema and residual fluid rather than as a comprehensive vascular or compartment evaluation.

The approximately 5-mm skin opening was approximated with adhesive skin-closure strips. No skin necrosis, recurrent ischemic change, significant bleeding, sensory or motor deficit, or functional loss developed during the observed follow-up. At seven-day follow-up, the local findings had completely resolved.

No clinical photographs were obtained during the acute event, and the ultrasound images were not saved; therefore, no clinical or imaging documentation was available for publication. The diagnosis was clinical, and the 24-hour ultrasound was obtained for follow-up assessment rather than to establish the diagnosis.

The patient provided verbal assent to the publication of the case. Written informed consent for open-access publication of this anonymized case report was obtained from her parents. According to institutional policy, an anonymized single-patient case report does not require review or approval by the institutional research ethics committee, nor is a formal exemption or waiver required. Informed consent for publication is the required ethical safeguard for this type of report. Accordingly, no separate ethics committee submission or formal exemption was sought.

## Discussion

The clinical findings were concerning for early perfusion compromise but did not establish a diagnosis of acute compartment syndrome. Compartment pressures were not measured. Importantly, preserved distal pulses and intact motor and sensory function do not exclude early compartment syndrome; these findings have been documented in propofol-associated compartment syndrome [6]. Accordingly, the presentation is described conservatively as impending tissue ischemia or early local perfusion compromise. This terminology is consistent with pediatric extravasation guidance, which identifies blanched skin and prolonged capillary refill as signs of impending tissue ischemia and recommends immediate surgical referral for neurovascular compromise or suspected compartment syndrome [9].

Initial steps followed conventional extravasation principles. Administration was stopped, and aspiration was attempted through the still-indwelling catheter before its removal; no return was obtained. The subsequent five 22-gauge needles were inserted to approximately 1 cm and used solely as passive drainage pathways, without negative-pressure aspiration. They produced no drainage or reduction in tissue tension. A 16-gauge needle was subsequently advanced approximately 3 cm through the pre-existing catheter tract. Negative-pressure aspiration through this needle was unsuccessful, and passive drainage after its removal also yielded no fluid.

These punctures were empirical drainage attempts rather than a guideline-described saline washout. Importantly, a pediatric surgeon was present during the event and suggested the subsequent limited blunt opening of the pre-existing tract, so contemporaneous surgical input was available when the maneuver was chosen. Only after the Klemmer clamp had been introduced approximately 5 mm into the tract and gently opened in multiple radial directions did visible lipid-containing fluid drain and tissue tension resolve. Gentle external expression was used only after an egress pathway had opened. Pediatric guidance advises against pressure during initial extravasation management but does describe gentle milking during formal saline washout; the maneuver used here therefore should not be generalized from this single observation [9].

The sequence raises the possibility that the limiting step was not simply the diameter of the skin opening. The failure of both multiple passive needle punctures and linear tract enlargement with a substantially larger needle suggests that the simple creation of a larger opening was insufficient. One possible explanation is that the extravasated material had distributed within subcutaneous tissue planes that did not communicate effectively with the catheter tract or needle punctures. Limited multidirectional blunt spreading may have opened communication between these spaces and the surface, thereby creating a lower-resistance pathway for egress. This explanation remains speculative and cannot be established from a single case.

The physical evacuation of extravasated material is not itself novel. Gault described liposuction and saline flush-out approaches [10], and contemporary pediatric washout techniques use multiple punctures, subcutaneous saline irrigation, and gentle milking toward exit sites [9,11]. For radiographic contrast extravasation, multiple large-bore punctures, followed by squeezing and techniques using multiple slit or stab incisions, have also been reported [12-14]. These contrast-media reports are mechanistic analogies and should not be interpreted as evidence for the management of propofol extravasation. In severe propofol-associated compartment syndrome, fasciotomy has released retained propofol under pressure, followed by extensive irrigation or washout [6,7]. Collectively, these reports support only the broader concept that physical evacuation or decompression has been used in selected severe extravasations; the pediatric evidence base remains low quality, and comparative effectiveness among invasive techniques has not been established [11].

The final drainage maneuver in the present case was less extensive than these established approaches. It used the pre-existing catheter tract, no additional skin incision, no saline irrigation, no liposuction, and no fasciotomy. However, the overall management was not literally a single-tract procedure because five 22-gauge drainage punctures had already been attempted. No previous published description was identified of propofol extravasation managed by limited blunt opening of a pre-existing intravenous tract to permit direct evacuation without additional skin incisions, saline washout, or fasciotomy. Because this was not a systematic literature search, this novelty claim is necessarily provisional. The description of the maneuver in this report is therefore observational and should not be interpreted as defining a validated or standardized technique.

The anatomical location requires particular caution. The antecubital fossa contains superficial sensory nerves and veins, as well as deeper neurovascular structures. Blind instrumentation or radial spreading in this region carries intrinsic risks, including injury to superficial nerves, vascular laceration, bleeding, and trauma to deeper structures. Although the Klemmer clamp in this case was introduced only approximately 5 mm into the pre-existing tract and no significant bleeding or sensory, motor, or other neurovascular complication was observed, the absence of complications in a single patient does not establish procedural safety. The described maneuver should not be regarded as risk-free, routinely reproducible, or generally recommended. Any invasive intervention in this anatomical region requires the careful consideration of local anatomy and appropriate surgical involvement.

The apparently normal 5-mL saline flush also deserves caution. The absence of pain or visible swelling did not prove that the catheter tip was securely intravascular. Because the catheter had been placed previously on the ward, partial or complete displacement may already have occurred, although this cannot be established retrospectively. Similarly, the relatively high baseline resistance of manual injection through a 24-gauge catheter may have made a change in resistance difficult to appreciate, but the case does not establish this as the mechanism of delayed recognition.

The patient remained awake and fully responsive without evident systemic sedation after the initial 100-mg propofol administration. This observation reinforces the uncertainty regarding the fraction of the dose that remained intravascular versus the amount that entered the tissues. Although 100 mg was administered through the original catheter, the case does not permit the quantification of the actual extravasated propofol volume or dose. Likewise, the estimated 8-10 mL recovered after blunt opening included blood and tissue fluid and cannot be equated with the amount of propofol originally extravasated.

The 24-hour ultrasound also had important limitations. It confirmed soft-tissue edema without a significant residual collection but was not a comprehensive vascular or compartment study. Neither color nor power Doppler evaluation was performed, venous patency or thrombosis was not specifically documented, and a dedicated examination of the deeper fascial compartments was not recorded. The favorable clinical evolution therefore rests primarily on serial clinical findings rather than on imaging evidence of preserved vascular patency or normal compartment anatomy.

This report has additional important limitations. It is a single uncontrolled observation; neither the actual extravasated volume nor the recovered volume was measured; no standardized extravasation severity score or objective percentage of limb swelling was documented; no tissue or compartment pressure measurements were obtained, and the favorable outcome cannot be attributed causally to the intervention. Because general anesthesia was induced before the subsequent drainage attempts, the serial assessment of pain and pain with passive stretch was no longer possible during the intervention.

The absence of clinical photographs and saved ultrasound images materially limits the independent verification of both the initial physical findings and the response to treatment. Follow-up was limited to seven days, so late scar, sensory, or functional sequelae were not assessed. Although a pediatric surgeon was present and suggested the final maneuver, the blunt instrumentation of an intravenous catheter tract is not guideline-standard and could cause vascular or nerve injury, bleeding, infection, or additional tissue trauma.

Guideline-described alternatives, including saline washout with or without hyaluronidase in selected pediatric extravasations, were not used, and this case cannot compare the described maneuver to those approaches [9,11]. Suspected compartment syndrome or ongoing neurovascular compromise requires urgent surgical assessment and should not be delayed by attempts at local drainage [6,9].

The present observation therefore supports only a hypothesis: in a rapidly recognized, tense propofol extravasation with early perfusion compromise and failed catheter aspiration, prompt physical evacuation may be mechanistically useful in selected circumstances. Whether drainage through a pre-existing tract can be performed safely or offers any advantage over established washout strategies requires further study.

## Conclusions

An adolescent developed a tense antecubital propofol extravasation during the manual administration of 10 mL of propofol 1%, with pallor and delayed capillary refill despite a palpable radial pulse and preserved neurologic function. Although 100 mg of propofol was administered through the original catheter, the amount actually entering the tissues could not be determined. The patient remained fully conscious without evident systemic sedative effects. Catheter aspiration, multiple passive needle drainage attempts, and aspiration and linear tract enlargement with a larger needle were ineffective. The limited blunt opening of the pre-existing catheter tract was temporally followed by the drainage of whitish lipid-containing fluid, rapid tissue softening, and improvement in perfusion, with complete clinical resolution by seven days.

This single case does not establish efficacy or safety, does not define the amount of propofol actually extravasated, and cannot determine whether the observed outcome differed from what would have occurred with guideline-described washout or conservative management. The blind instrumentation of the antecubital region also carries potentially important neurovascular risks. The observation is hypothesis-generating and may justify further study of prompt physical evacuation in selected tense propofol extravasations with early perfusion compromise, but it does not support the routine adoption of the described maneuver.
